# Association of Single Nucleotide Polymorphism in the DGAT1 Gene with the Fatty Acid Composition of Cows Milked Once and Twice a Day

**DOI:** 10.3390/genes14030767

**Published:** 2023-03-21

**Authors:** Inthujaa Sanjayaranj, Alastair K. H. MacGibbon, Stephen E. Holroyd, Patrick W. M. Janssen, Hugh T. Blair, Nicolas Lopez-Villalobos

**Affiliations:** 1 Animal Science, School of Agriculture and Environment, Massey University, Private Bag 11 222, Palmerston North 4442, New Zealand; 2 Department of Animal Science, Faculty of Agriculture, Eastern University, Chenkaladi, Batticaloa 30000, Sri Lanka; 3 Fonterra Research and Development Centre, Private Bag 11029, Palmerston North 4442, New Zealand; 4 School of Food and Advanced Technology, Massey University, Private Bag 11 222, Palmerston North 4442, New Zealand

**Keywords:** dairy cattle, *DGAT1*, fatty acids, milking frequency, SNP genotype

## Abstract

A single nucleotide polymorphism (SNP) rs109421300 of the diacylglycerol acyltransferase 1 (*DGAT1*) on bovine chromosome 14 is associated with fat yield, fat percentage, and protein percentage. This study aimed to investigate the effect of SNP rs109421300 on production traits and the fatty acid composition of milk from cows milked once a day (OAD) and twice a day (TAD) under New Zealand grazing conditions. Between September 2020 and March 2021, 232 cows from a OAD herd and 182 cows from a TAD herd were genotyped. The CC genotype of SNP rs109421300 was associated with significantly (*p* < 0.05) higher fat yield, fat percentage, and protein percentage, and lower milk and protein yields in both milking frequencies. The CC genotype was also associated with significantly (*p* < 0.05) higher proportions of C16:0 and C18:0, higher predicted solid fat content at 10 °C (SFC_10_), and lower proportions of C4:0 and C18:1 *cis*-9 in both milking frequencies. The association of SNP with fatty acids was similar in both milking frequencies, with differences in magnitudes. The SFC_10_ of cows milked OAD was lower than cows milked TAD for all three SNP genotypes suggesting the suitability of OAD milk for producing easily spreadable butter. These results demonstrate that selecting cows with the CC genotype is beneficial for New Zealand dairy farmers with the current payment system, however, this would likely result in less spreadable butter.

## 1. Introduction

In New Zealand, twice-a-day (TAD) milking is the standard milking method of milk harvest. However, in the late 1990s farmers started to adopt once-a-day (OAD) milking due to several benefits such as reduced labour cost, improved labour efficiency, improved health and reproductive performance of cows, and additional employment opportunities to farmers [1,2,3]. OAD milk tends to reduce the milk yield and increase the concentration of fat and protein compared to TAD milking [2,4,5].

When considering milk composition, fat is an important component that plays an important role in the New Zealand milk payment scheme [6] and the processability of milk [7]. Bovine milk fat is made up of 98% triglycerides [8]. Triglycerides consist of glycerol and three fatty acids and are mainly synthesised from diglycerides and this step is catalysed by diacylglycerol acyltransferase enzymes (*DGAT1* and *DGAT2*). Fatty acids in milk are derived from two main sources: the short- and medium-chain fatty acids are produced by de novo synthesis and long-chain fatty acids are absorbed directly from the diet [9]. Fatty acid composition is also affected by breed [10,11,12], stage of lactation [13,14], diet [15,16] and season [17,18]. Another important factor influencing the milk fatty acid composition within the breed is genetic variation. The fatty acid composition of milk is also considered important in influencing the processability of milk, especially butter making. The fatty acid composition of the milk influences the butter hardness by affecting the solid fat content at 10 °C (SFC_10_) [19]. Butter hardness reflects the ease with which the butter can be spread on bread [20].

Recently, one of the dairy industry’s main interests is to understand cows’ genetic variation for desired traits to make a better selection process. The fatty acid composition of cow milk is one trait that is widely studied [10,21,22]. Several studies have reported the effect of the diacylglycerol acyltransferase 1 (*DGAT1*) polymorphism in the bovine genome on major milk traits of cows [23,24,25]. Some studies have also reported the effect of *DGAT1* polymorphism on the fatty acid composition of milk [25,26,27]. However, there are some other polymorphisms in proximity to *DGAT1* genes that could contribute to the variation in the fatty acid composition of cow milk as the quantitative trait locus (QTL) which has a major effect on milk production traits is positioned at the centromere end of chromosome 14 where the *DGAT1* gene is located. The association of the single nucleotide polymorphism (SNP; rs109421300) of *DGAT1* with fat and protein percentages in cows milked once a day (OAD) and twice a day (TAD) has been reported by [28]. However, the effect of this SNP on the milk fatty acid composition has not been tested yet. This study aimed to investigate the effect of SNP rs109421300 on the fatty acid composition of cows milked OAD and TAD.

## 2. Materials and Methods

### 2.1. Farm and Feeding

Cows on the Massey University No.1 dairy farm were fed ryegrass (*Lolium perenne*) and white clover (*Trifolium repens*) pasture with a low level of supplementation and were milked OAD in the mornings. The stocking rate of the No. 1 Dairy was 2.4 cows/ha. Cows on the Massey University No. 4 dairy farm were fed ryegrass and white clover pasture with a high level of supplementation and were milked TAD in the mornings and afternoons. The stocking rate of the No. 4 Dairy was 2.6 cows/ha. The feed allocation and the chemical composition of feed given to cows on both farms in the milking season 2020–2021 are presented in Table 1.

### 2.2. Cows and Milk Sampling

A herd from No. 1 Dairy (OAD milking herd), comprised 232 cows with a breed proportion of 70 Holstein Friesian (F), 104 Holstein Friesian × Jersey (F × J), and 58 Jersey (J), and another herd from No. 4 Dairy (TAD milking herd) comprised of 182 cows with the breed proportion of 102 F and 80 F × J were selected for this study. The two herds contained primiparous (No. 1 Dairy = 55 and No. 4 Dairy = 118) and multiparous (No. 1 Dairy = 177 and No. 4 Dairy = 64) cows. The cows from both herds were free of clinical mastitis and metabolic diseases during the sampling period. The milk samples were collected during early (September 2020), mid (December 2020), and late (March 2021) lactation in the milking season 2020–2021. The cows had a minimum of three herd-test records for the milking season. Milk samples were collected using Waikato milk flow meters. The samples were stored in the refrigerator at 0–4 °C immediately after collection without adding any preservatives and were analysed for milk composition within two days of collection.

### 2.3. Determination of Milk Composition and Fatty Acid Composition

Using standard calibration equations, all samples were analysed for fat, protein, and lactose percentages by a Milkoscan FT1 (Foss, Hillerød, Denmark). Calibration equations for individual fatty acids were developed using the FTIR calibrator software (Foss Analytical, Hillerød, Denmark) with the reference values obtained from gas chromatography (GC) [29] with Shimadzu GC-2010 plus. The proportions of individual fatty acids were predicted using the calibration equations in Milkoscan FT1 (Foss, Hillerød, Denmark).

### 2.4. Genotyping

DNA extracted from ear punch tissue samples were genotyped using Bovine Illumina 50K SNP chips. The analysis used all the animals that met the call rate of 80%. The SNP rs109421300 was selected for the statistical analysis because of the proximity to the *DGAT1* gene and the significant effect on fat percentage.

### 2.5. Statistical Analysis

Statistical analysis was performed using SAS version 9.4 (SAS Institute Inc., Cary, NC, USA). Least square means and standard errors for the study traits were obtained using the PROC MIXED procedure with the following linear mixed model:

Yijklmn = μ + Mi + Bj(Mi) + Lk + Sl + GMmi + + β1dn + Cn + eijklmn

where

Yijklmn is the observation for any of the production and composition traits in milking frequency i, breed j, lactation number k, stage of lactation l, genotypes m, and cow n.

μ is the population mean.

Mi is the fixed effect of milking frequency (i = OAD and TAD).

Bj (Mi) is the fixed effect of breed j nested in milking frequency i (j = F, F × J, and J).

Lk is the fixed effect of lactation number (k = 1, 2, …5).

Sl is the fixed effect of the stage of lactation (l = early, mid, and late).

GMmi is the fixed effect of interaction between genotype m and milking frequency i.

β1 is the regression coefficient of the linear effect of deviation (dn; days) from herd median calving date on trait Y of cow n.

Cn is the random effect of cow (n = 1,2, …, 414) assumed with mean zero and variance $\sigma_{c}^{2}$.

eijklmn is the residual random error assumed with mean zero and variance $\sigma_{e}^{2}$.

A limitation of this study was the confounding effect of feed and milking frequency. The feed provided for cows on each farm was slightly different. Cows milked TAD were provided higher supplements compared to cows milked OAD. The genetic merit of the cows on both farms was similar as they were from the same few sires. The breed was nested in milking frequency as the two milking frequencies had different breed proportions: F, F × J, and J cows in OAD milking and F and F × J in TAD milking.

Solid fat content at 10 °C was predicted from an equation developed using PROC REG. The prediction equation was developed using the fatty acid composition and SFC_10_ data from the study of MacGibbon [19].

Using the same PROC MIXED model, the effects of SNP genotypes on fatty acids C4:0, C16:0 and C18:1 *cis*-9, and SFC_10_ at different stages of lactation in cows milked OAD and TAD were estimated by including the interaction between SNP genotypes, milking frequency and stages of lactation as a fixed effect.

Partial correlation coefficients (adjusted by the factors in the model) between the study traits were obtained using MANOVA. The SNP variances for the traits were obtained by fitting the SNP rs109421300 as a random effect in the model.

## 3. Results

The frequency of genotypes CC, CT, and TT were 31.9, 44.2, and 23.9%, respectively, in the OAD herd, and 19.8, 52.1, and 28.1%, respectively, in the TAD herd. The frequency of the C and T alleles were 54 and 46%, respectively, in the OAD herd while the frequencies were 46 and 54% in the TAD herd, respectively. The *p*-values for the chi-square test for the OAD herd and TAD herd were 0.1001 and 0.524, respectively. The descriptive statistics for the production traits and composition traits of cows milked OAD and TAD are presented in Table 2.

**Figure 1:**
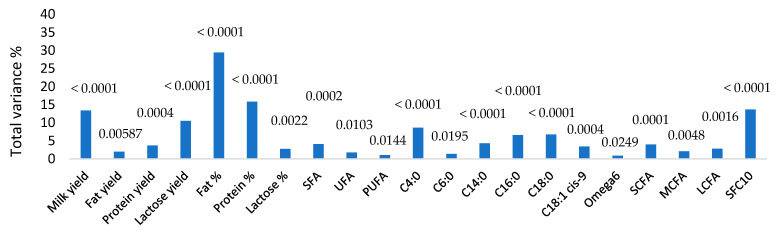
The proportions of variance explained by the SNP rs109421300 for the production traits and composition traits in cows milked in the milking season 2020-2021. SFA = Saturated fatty acids; UFA = Unsaturated fatty acids; PUFA = Polyunsaturated fatty acids; SCFA = Short-chain fatty acids (sum of C4:0, C6:0, and C8:0); MCFA = Medium-chain fatty acids (sum of C10:0, C12:0); LCFA = Long-chain fatty acids (sum of C14:0, C16:0, C18:0 and C18:1 *cis*-9, and omega-6); SFC_10_ = Solid fat content at 10 °C.

Figure 1 shows the proportion of variance explained by the selected SNP rs109421300 and the probability values for the variance. The SNP explained approximately 30% of the variance for fat percentage, 15% for protein percentage, and 13% for milk yield. For fatty acids, approximately 10% of the variance for C4:0 was explained by the SNP and 6% for C16:0 and C18:0. The SNP did not control a significant proportion of variance for fatty acids C8:0, C10:0, and C12:0. Approximately 14% of the variance was explained by the SNP for the variable SFC_10_.

The partial correlation coefficients between fat percentage and fatty acid composition of milk are shown in Table 3. In both milking frequencies, the correlations between fat and fatty acids were weak to moderate. The correlations between fat percentage and fatty acids from C4:0 to C12:0 were negative for cows milked TAD, but were not significant for cows milked OAD except for C4:0 and C6:0. Correlation between fat and C16:0, and fat and C18:1 *cis*-9 were week and positive in both milking frequencies. In cows milked TAD the correlation between fat and solid fat content was significant and positive.

Least-squares means and standard errors for production and composition traits for the three SNP rs109421300 genotypes in both milking frequencies are shown in Table 4. Compared with the CC genotype, the TT genotype was significantly (*p* < 0.05) associated with higher milk yield, protein yield, and lactose yield in both milking frequencies whereas the CC genotype was associated with significantly (*p* < 0.05) higher values for fat yield, fat percentage, and protein percentage compared to the TT genotype in both milking frequencies. The CT genotype was intermediate for all these traits. In cows milked OAD and TAD, the TT genotype was associated with significantly (*p* < 0.05) higher proportions of C4:0 and C18:1 *cis*-9 compared to the CC genotype whereas the CC genotype was associated with significantly higher proportions of C16:0 and C18:0 compared to the TT genotype. The CT had intermediate values for the proportions of C16:0 and C18:0.

When comparing the two milking frequencies, all three genotypes of cows milked OAD showed significantly lower values for the production traits and significantly higher values for the percentages of fat and protein, compared to the corresponding genotypes of cows milked TAD. In the case of fatty acids C4:0 and C18:1 *cis*-9, cows milked OAD showed significantly lower proportions for all three genotypes than cows milked TAD.

In cows milked OAD, the CC genotype was associated with significantly higher income for milk solids while in cows milked TAD, the genotypes did not significantly differ. The variation in milk income between the two milking frequencies was not significant. Higher SFC_10_ was linked to the CC genotype compared to the TT genotype in cows milked OAD and TAD. The genotype CT was intermediate for SFC_10_. When comparing the CC genotype in cows milked TAD, the CC genotype in cows milked OAD was associated with lower SFC_10_. 

**Figure 2:**
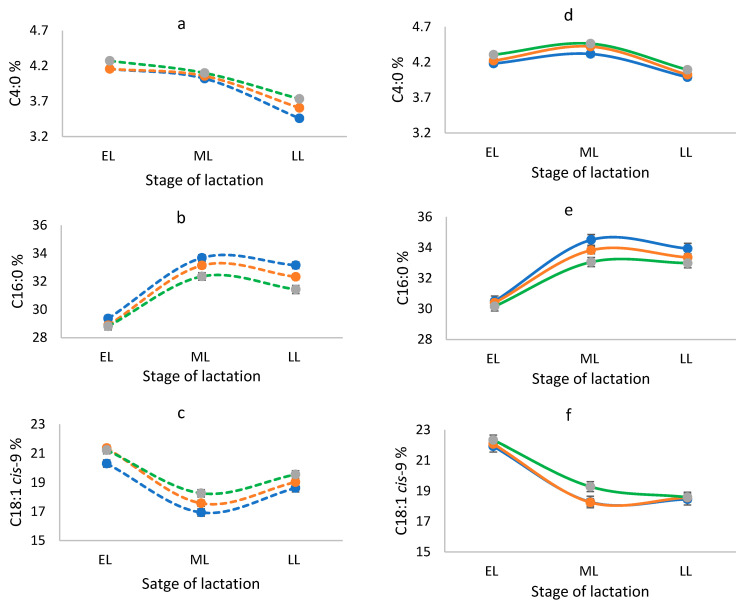
Proportions of (**a**) C4:0, (**b**) C16:0, and (**c**) C18:1 *cis*-9 for genotypes CC (….), CT (**….**), and TT (**….**) in cows milked once a day, and proportions of (**d**) C4:0, (**e**) C16:0, and (**f**) C18:1 *cis*-9 for genotypes CC (**—**), CT (**—**) and TT (**—**) in cows milked twice a day during the production season 2020–2021. EL = early lactation (<90 days); ML = mid lactation (90–180 days); LL = late lactation (>180 days). The vertical bars show standard errors.

**Figure 3:**
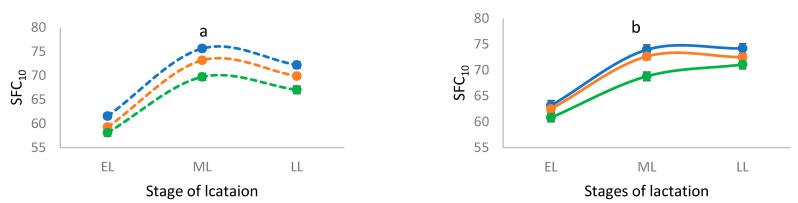
Solid fat content at 10 °C (SFC_10_) for genotypes CC (….), CT (**….**), and TT (**….**) in cows milked once a day (**a**), and for genotypes CC (**—**), CT (**—**) and TT (**—**) in cows milked twice a day (**b**) during the production season 2020–2021. EL = early lactation (<90 days); ML = mid lactation (90–180 days); LL = late lactation (>180 days). The vertical bars show standard errors.

The three genotypes showed similar patterns throughout the lactation in both milking frequencies with significant differences in mid and late-lactation stages. In early lactation, the proportions of C4:0 and C18:1 *cis*-9 were lower and the proportion of C16:0 was higher compared to mid- and late-lactation in both milking frequencies (Figure 2). The SFC_10_ was also lower in early lactation in both milking frequencies (Figure 3). The genotype TT showed lower SFC_10_ throughout the lactation.

## 4. Discussion

The *p*-values (*p* < 0.05) from the Chi-square test suggest that the population was at equilibrium [30]. The *DGAT1* gene is widely accepted to have the most significant effect on milk production traits [23,26,31]. The SNP rs109421300 selected in this study is located on chromosome 14 in the *DGAT1* region at the 1801116 bp position [32]. Lopez-Villalobos et al. [28] reported a significant association of the SNP with the fat percentage in cows milked OAD and TAD. Other SNPs in the proximity to the *DGAT1* gene were tested for the association for fat percentage and the SNP rs109421300 showed the highest association with fat percentage. In this study, the SNP was significantly associated with the fatty acid composition and SFC_10_ of cows milked OAD and TAD.

Few studies have reported the significant effect of the SNP rs109421300 on the milk production traits of cows milked OAD and TAD [28,31]. This SNP has antagonistic pleiotropy between fat yield and milk yield, and fat yield and protein yield [31]. The current study reported the same antagonistic effect in cows milked OAD, with the CC genotype being significantly associated with higher fat yield and lower milk and protein yield. The SNP was positively associated with lower milk and protein yields with no effect on the fat yield in TAD milking. This could be due to the moderate correlation coefficient between fat yield and fat percentage which was 0.43 and is in agreement with Schennink et al. [26] in Dutch Holstein Friesian cows, and with Lopez-Villalobos et al. [33] in F, F × J, and J cows. In cows milked OAD the correlation coefficient between fat yield and fat percentage was 0.94. The CC genotype was also associated with a significant positive effect on the fat percentage in both milking frequencies, which is also in agreement with Jiang et al. [31] and Lopez-Villalobos et al. [28]. This could be due to the large negative effect of this SNP on milk yield and the large positive effect on fat yield [31]. This partially agrees with the results of this study, as considerable variation was explained by the SNP for milk yield. Overall, the effect of SNP rs109421300 between fat yield and milk yield was antagonistic, which resulted in a strong and positive effect on fat percentage.

As the SNP rs109421300 was highly associated with fat percentage, the partial correlations between fat and individual fatty acids were analysed. In cows milked TAD, the correlation coefficients between de novo synthesised fatty acids (C4:0–C12:0) were negative and weak to moderate. This is partially in agreement with Schennink et al. [26], who reported positive and weak correlations, and Lopez-Villalobos et al. [33], who reported positive and moderated correlations. The positive correlation between fat percentage and C16:0 is in agreement with Schennink et al. [26], Lopez-Villalobos et al. [33], and Soyeurt et al. [34], but the correlations were stronger (0.88, 0.43 and 0.86, respectively) in these studies. The positive and weak correlation between fat percentage and C18:0 is in agreement with Schennink et al. [26], and in contradiction with Lopez-Villalobos et al. [33] and Soyeurt et al. [34], who reported strong positive and moderate negative correlations, respectively. The positive correlation between fat percentage and C18:1 *cis*-9 is in agreement with Soyeurt et al. [34] where the correlation coefficient was higher (0.66) than in the present study. In contrast, Schennink et al. [26] and Lopez-Villalobos et al. [33] reported negative correlations. The positive and significant correlation between fat percentage and SFC_10_ in cows milked TAD is in agreement with MacGibbon, [19] who reported a positive correlation (0.78). The reason for differences in the correlation coefficients in the above study could be the variation in breed, feed, and management practices.

There are no studies estimating the correlation coefficients between fat percentage and individual fatty acids in milk from cows milked OAD. In the present study, the correlations between fat and fatty acids were weak to moderate. Correlations were in the same directions but in different magnitudes, for fatty acids, C4:0, C6:0, C8:0, C10:0, C12:0, C18:0, SCFA, and SFC_10_ in cows milked OAD and TAD. The correlation coefficients were slightly lower for OAD milking compared to TAD milking frequency. In this study, the correlation coefficients between fat percentage and fatty acids suggest that the SNP could have a less significant effect on individual fatty acids, especially de novo synthesis fatty acids compared to the effect on fat percentage. This is also justified by the low and no variance determined for the SNP for these fatty acids except for C4:0. This would be due to the higher affinity of *DGAT1* to butyryl-CoA and palmitoyl-CoA [35]. Estimating the genetic variance components for the study traits may explain more about the association of SNP with the traits [36] as estimates of genetic correlations and heritabilities for fatty acids would be helpful in finding the extent of the same gene affecting different traits.

The CC genotype of the SNP was positively associated with the proportions of C16:0 and C18:0, and negatively associated with the proportion of C4:0, C18:1 *cis*-9, and short-chain fatty acids in both milking frequencies. This suggests that selecting cows for higher fat yield for the SNP, will lead to a correlated increase in the proportions of C16:0 and C18:0 and a decrease in the proportions of C18:1 *cis*-9 and short-chain fatty acids. This is not optimum for the processability of milk, especially for making butter. Milk with these fatty acids in the above-mentioned proportions would lead to less spreadable butter [19,37]. This is also explained by the higher SFC_10_ for the CC genotype, which would tend to produce less spreadable butter. Higher SFC_10_ leads to lower spreadability of butter [19,37]. Generally, household consumers prefer softer butter, but some food processing companies, for example, the pastry industry, prefer harder butter to maintain flakiness and crispiness [38]. In New Zealand, cows are selected mainly for higher fat and protein yields. Therefore, selecting cows for the CC genotype would be beneficial for higher income but not for making butter.

In this study, the SNP was not significantly associated with the proportion of de novo synthesised fatty acids in both milking frequencies (the effect was very low for proportions of C6:0 and C10:0 for TAD milking). This may be due to the same origin of these fatty acids and similar mechanisms involved in synthesis in the mammary gland. Soyeurt et al. [34] reported high genetic correlations between these fatty acids and explained that the reason could be the similarities in their origin. Knutson et al. [22] reported that some genes in chromosomes 11, 13, 17, and 19 were highly correlated with de novo synthesised short- and medium-chain fatty acids (C4:0–C14:0) in cows milked TAD. Overall, the present study reports although the SNP is highly linked to fat percentage, the association was not stronger for fatty acids. Future research with genome-wide association studies (GWAS) would be helpful in finding suitable chromosomes, genes, and SNPs associated with milk composition in cows milked OAD.

The directions of SNP genotypes for most of the traits in this study were similar across milking frequencies. However, the magnitude of the genotypes for some traits behaved differently. The milk yield, fat yield, protein yield, and lactose yield were lower, and fat and protein percentages were higher for all three SNP genotypes in cows milked OAD compared to cows milked TAD. Apparently, OAD milking reduces milk production and modifies milk composition. Similarly, the proportions of de novo synthesis fatty acids, mainly C6:0–C14:0, were higher, and proportions of long-chain fatty acids were lower in cows milked OAD for all three genotypes compared to cows milked TAD. Similar results were reported by Sanjayaranj et al. [39] in the previous work comparing fatty acid composition between cows milked OAD and TAD.

The variation in the magnitude of the same genotype in different milking frequencies could be due to the interaction between the genotype and the environment. Falconer and Mackay [40] reported that animals would perform differently in diverse environments due to the genotype-by-environment (G × E) interaction. Lopez-Villalobos [41] reported that variation in traits is caused by the effect of genes, environment, and G × E interaction. In this study, the expression of SNP genotypes was affected by the milking frequency. The interaction of milking frequency with the mechanism of fatty acid synthesis and the concentration of precursors for fatty acid synthesis [42] could be the factors affecting the expressions of SNP genotypes in different milking frequencies.

Expression of the *DGAT1* gene is also affected by the season and stages of lactation [43,44]. In New Zealand, the season and stage of lactation are considered important factors affecting the processability of milk [45]. In this study, all three genotypes showed similar patterns throughout lactation for the fatty acids C4:0., C16:0, C18:1 *cis*-9 and SFC_10_ in both milking frequencies. The proportions of these fatty acids and SFC_10_ reveal that butter produced in early lactation tends to be easily spreadable, especially with the TT genotype. This is in agreement with Auldist et al. [45] who reported that the SFC_10_ was lower in early lactation milk compared to milk from the other two stages of lactation. The trends of the curves also show that the spreadability of the butter would tend to decrease in the mid- and late-lactation stages, with the TT genotype more likely to produce easily spreadable butter compared to other genotypes. However, these changes are smaller than the overall seasonal changes. In New Zealand, the stage of lactation is synchronized with the season, pasture quality and feed availability [18]. Therefore, it is difficult to determine the actual influence of the stage of lactation on the expression of SNP for fatty acids. Auldist et al. [45] reported that the effect of the season was greater than the effect of the stage of lactation. The effect of season was not explored in this study. This would need to be confirmed by assessment of the actual product produced.

## 5. Conclusions

The CC genotype of SNP rs109421300 was largely and positively associated with fat yield, fat percentage, and protein percentage, and negatively associated with milk and protein yields. The associations of SNP genotypes with fatty acids were similar in both milking frequencies, however, the magnitudes were different due to the differences in the environment. The SNP also had a lower association with the de novo synthesised fatty acids in both milking frequencies. The CC genotype was associated with higher proportions of C16:0 and C18:0, lower proportions of C4:0 and C18:1 *cis*-9, and higher SFC_10_ in cows milked OAD and TAD, suggesting selecting cows with the CC genotype would lead to the production of butter that would be less spreadable. The CC genotype in cows milked OAD produced lower proportions of C16:0 and C18:0, and lower SFC_10_ compared to the same genotype in cows milked TAD. Selecting cows with the CC genotype of SNP rs109421300 could be beneficial for New Zealand dairy farmers with the current payment system while selecting cows with the TT genotype, especially in OAD milking would be beneficial only for butter making. The results of this study pave the way for researchers, farmers, and processing companies to think of a possible way of producing more spreadable butter with OAD milking.

## Figures and Tables

**Table 1:** Feed allocation and chemical composition of diet provided at the No. 1 Dairy and No. 4 Dairy during the sampling period in the milking season 2020–2021.

**Farm**	**No. 1 Dairy (OAD ^12^)**	**No. 4 Dairy (TAD ^13^)**
Feed allocation (kg DM per cow per day)		
Pasture ^1^	8.2	14.7
Herb mix crop ^2^	3.7	-
Maize silage	1.0	4.0
DDG ^3^	1.5	1.0
Tapioca pellets	0.8	-
Concentrate ^4^	2.0	2.5
Dry roughage ^5^	-	0.6
Baleage ^6^	4.0	5.5
Feed chemical composition		
ME ^7^ MJ/kg DM	11.33	11.07
CP ^8^ g/100 g DM	20.49	19.95
NDF ^9^ g/100 g DM	38.91	44.43
ADF ^10^ g/100 g DM	22.44	23.24
SSS ^11^ g/100 g DM	13.22	15.09
Lipid g/100 g DM	4.44	4.42

^1^ Comprised perennial ryegrass (*L.p)* and white clover (*T.r*); ^2^ comprised plantain (*Plantago lanceolata*), chicory (*Cichorium intybus*), and red clover (*Trifolium pratense*); ^3^ distilled dried grain; ^4^ grain-based concentrate; ^5^ hay and straw; ^6^ Lucerne baleage in No. 1 Dairy and grass baleage in No. 4 Dairy; ^7^ metabolisable energy; ^8^ crude protein; ^9^ neutral detergent fibre; ^10^ acid detergent fibre; ^11^ SSS = soluble sugars and starch. DM = dry matter; ^12^ once-a-day milking; ^13^ twice-a-day milking.

**Table 2:** Descriptive statistics of production traits, gross milk composition, and fatty acid composition of cows milked once a day (OAD) and twice a day (TAD) during the 2020–2021 production season.

**Milking Frequency**	**OAD**						**TAD**					
**Variable**	N	Mean	SD	CV%	Min	Max	N	Mean	SD	CV%	Min	Max
Milk yield (L/cow/day)	668	17.89	6.3	35.2	4.6	39.9	543	19.50	5.56	28.5	6.40	39.50
Fat yield (kg/cow/day)	668	0.91	0.32	35.4	0.26	5.22	543	0.88	0.22	24.6	0.31	1.92
Protein yield (kg/cow/day)	668	0.73	0.22	30.3	0.21	1.48	543	0.71	0.17	23.6	0.29	1.54
Lactose yield (kg/cow/day)	668	0.87	0.33	38	0.19	2.13	543	0.99	0.30	30.5	0.29	2.08
Fat percentage	694	4.86	1.04	21.3	1.72	8.53	543	3.98	0.85	21.4	1.27	6.89
Protein percentage	694	4.14	0.4	9.6	3.19	5.67	543	3.76	0.37	9.9	2.74	5.01
Lactose percentage	694	4.83	0.26	5.3	3.7	5.43	543	5.03	0.24	4.7	4.29	5.51
Fatty acid (% of the total FA)												
SFA ^1^	694	70.65	3.31	4.7	51.57	81.78	543	70.08	2.96	4.2	58.64	77.94
UFA ^2^	694	29.85	2.68	9	21.71	43.96	543	29.94	2.54	8.5	23.70	38.84
PUFA ^3^	694	3.13	0.52	16.7	2.01	4.61	543	2.87	0.41	14.5	1.49	3.85
C4:0	694	3.95	0.34	8.7	2.5	4.89	543	4.11	0.25	6.0	2.52	4.65
C6:0	694	2.87	0.25	8.7	1.37	3.49	543	2.83	0.20	6.9	1.71	3.29
C8:0	694	1.6	0.17	10.7	0.56	2.05	543	1.50	0.12	8.3	0.68	1.82
C10:0	694	3.7	0.46	12.3	0.93	4.93	543	3.33	0.34	10.3	1.19	4.38
C12:0	694	4.08	0.37	9.1	1.9	5.17	543	3.72	0.29	7.7	2.46	4.46
C14:0	693	13.07	1.35	10.3	5.44	16.05	543	12.58	1.22	9.7	7.14	15.27
C16:0	694	31.52	2.83	9	22.86	38.69	543	32.10	2.47	7.7	25.17	39.81
C18:0	694	12.62	1.59	12.6	7.87	22.23	543	13.28	1.22	9.2	9.94	21.37
C18:1 *cis*-9	694	19.18	2.76	14.4	11.81	37.88	543	20.54	2.69	13.1	14.75	31.99
Omega-6	694	1.74	0.51	29.5	0.47	4.03	543	1.55	0.31	20.1	0.37	2.28
SCFA ^4^	694	8.41	0.72	8.6	4.43	10.02	543	8.44	0.53	6.3	5.37	9.71
MCFA ^5^	694	20.83	1.91	9.2	8.64	25.43	543	19.63	1.64	8.4	11.57	23.37
LCFA ^6^	694	65.06	2.38	3.7	58.25	79.77	543	67.46	2.41	3.6	62.12	82.02
Milk income (NZD/cow/day)	668	14.8	4.64	31.3	4.26	57.33	543	14.34	3.37	23.5	6.27	31.16
SFC_10_^7^	694	67.53	8.6	12.7	17.7	89.57	543	67.44	7.13	10.6	41.03	86.96

^1^ Saturated fatty acids; ^2^ Unsaturated fatty acids; ^3^ Polyunsaturated fatty acids; ^4^ Short-chain fatty acids (sum of C4:0, C6:0 and C8:0); ^5^ Medium-chain fatty acids (sum of C10:0, C12:0, C14:0); ^6^ Long-chain fatty acids (sum of C16:0, C18:0 and C18:1 *cis*-9 and omega-6); Milk income was calculated using the equation NZD 9.78 × kg fat yield + NZD 8.89 × kg protein yield − NZD 0.033 × kg milk yield; ^7^ Solid fat content at 10 °C; N is from 3 sampling period (early, mid, and late lactation).

**Table 3:** Partial correlation coefficients between fat percentage and fatty acid composition of cows milked once a day (OAD) and twice a day (TAD).

Fatty Acids	Milking Frequency			
	OAD	*p*-Value	TAD	*p*-Value
SFA ^1^	−0.09	0.0228	−0.04	0.3954
UFA ^2^	0.2	<0.0001	0.14	0.0027
PUFA ^3^	−0.12	0.0022	−0.11	0.0117
C4:0	−0.15	<0.0001	−0.43	<0.0001
C6:0	−0.12	0.0017	−0.34	<0.0001
C8:0	0.01	0.8817	−0.20	<0.0001
C10:0	−0.02	0.5528	−0.18	<0.0001
C12:0	−0.01	0.7613	−0.11	0.0148
C14:0	−0.04	0.3424	0.04	0.3271
C16:0	0.24	<0.0001	0.32	<0.0001
C18:0	0.06	0.1455	0.23	<0.0001
C18:1 *cis*-9	0.17	<0.0001	0.11	0.0136
Omega6	−0.21	<0.0001	−0.21	<0.0001
SCFA ^4^	−0.13	0.0014	−0.38	<0.0001
MCFA ^5^	−0.03	0.4321	−0.03	0.5355
LCFA ^6^	0.37	<0.0001	0.45	<0.0001
SFC_10_ ^7^	0.03	0.5057	0.21	<0.0001

^1^ Saturated fatty acids; ^2^ Unsaturated fatty acid; ^3^ Polyunsaturated fatty acid; ^4^ Short-chain fatty acids (sum of C4:0, C6:0 and C8:0); ^5^ Medium-chain fatty acids (sum of C10:0, C12:0); ^6^ Long-chain fatty acids (sum of C14:0, C16:0, C18:0 and C18:1 *cis*-9 and omega-6); ^7^ Solid fat content at 10 °C.

**Table 4:** Least-squares means and standard errors for the association between milk production traits, milk composition, and fatty acid composition and SNP rs109421300 genotypes for cows milked once a day (OAD) and twice a day (TAD).

Variables	OAD			TAD			*p*-Value MF ^8^ ×SNP
	CC	CT	TT	CC	CT	TT	
Milk yield (L/cow/day)	16.57 ± 0.3 ^e^	18.23 ± 0.24 ^d^	18.83 ± 0.36 ^d^	22.17 ± 0.46 ^c^	23.56 ± 0.33 ^b^	25.00 ± 0.4 ^a^	<0.0001
Fat yield (kg/cow/day)	0.93 ± 0.02 ^b^	0.91 ± 0.01 ^b^	0.85 ± 0.02 ^c^	1.06 ± 0.03 ^a^	1.06 ± 0.02 ^a^	1.03 ± 0.02 ^a^	0.015
Protein yield (kg/cow/day)	0.70 ± 0.01 ^d^	0.75 ± 0.01 ^c^	0.75 ± 0.01 ^c^	0.84 ± 0.02 ^b^	0.87 ± 0.01 ^ab^	0.89 ± 0.02 ^a^	0.0025
Lactose yield (kg/cow/day)	0.80 ± 0.02 ^e^	0.89 ± 0.01 ^e^	0.91 ± 0.02 ^d^	1.13 ± 0.02 ^c^	1.19 ± 0.02 ^b^	1.25 ± 0.02 ^a^	<0.0001
Fat percentage	5.34 ± 0.07 ^a^	4.80 ± 0.05 ^b^	4.33 ± 0.08 ^c^	4.08 ± 0.1 ^c^	3.67 ± 0.07 ^d^	3.15 ± 0.09 ^e^	<0.0001
Protein percentage	4.27 ± 0.03 ^a^	4.15 ± 0.02 ^b^	4.05 ± 0.03 ^c^	3.84 ± 0.04 ^d^	3.74 ± 0.03 ^e^	3.60 ± 0.04 ^f^	<0.0001
Lactose percentage	4.80 ± 0.02 ^d^	4.85 ± 0.01 ^c^	4.79 ± 0.02 ^d^	5.07 ± 0.03 ^a^	5.04 ± 0.02 ^a^	4.97 ± 0.02 ^b^	0.0016
Fatty acid (% of the total FA)							
SFA ^1^	71.4 ± 0.21 ^a^	70.51 ± 0.17 ^bc^	70.1 ± 0.25 ^b^	71.55 ± 0.32 ^a^	71.42 ± 0.23 ^a^	70.88 ± 0.29 ^ac^	0.0016
UFA ^2^	29.29 ± 0.18 ^b^	29.9 ± 0.14 ^a^	30.18 ± 0.21 ^a^	28.53 ± 0.27 ^c^	28.59 ± 0.19 ^c^	28.79 ± 0.24 ^bc^	0.0171
PUFA ^3^	3.05 ± 0.03 ^b^	3.15 ± 0.02 ^a^	3.16 ± 0.03 ^a^	2.82 ± 0.04 ^c^	2.87 ± 0.03 ^c^	2.84 ± 0.04 ^c^	0.0291
C4:0	3.89 ± 0.02 ^e^	3.94 ± 0.02 ^d^	4.03 ± 0.02 ^c^	4.17 ± 0.03 ^b^	4.23 ± 0.02 ^b^	4.29 ± 0.03 ^a^	<0.0001
C6:0	2.85 ± 0.01 ^c^	2.86 ± 0.01 ^c^	2.89 ± 0.02 ^bc^	2.89 ± 0.02 ^bc^	2.93 ± 0.02 ^ab^	2.95 ± 0.02 ^a^	0.0543
C8:0	1.60 ± 0.01 ^a^	1.59 ± 0.01 ^ac^	1.59 ± 0.01 ^ac^	1.54 ± 0.01 ^b^	1.57 ± 0.01 ^bc^	1.56 ± 0.01 ^bc^	0.4008
C10:0	3.72 ± 0.03 ^a^	3.67 ± 0.02 ^a^	3.67 ± 0.03 ^a^	3.41 ± 0.04 ^c^	3.5 ± 0.03 ^b^	3.48 ± 0.04 ^bc^	0.1831
C12:0	4.12 ± 0.03 ^a^	4.05 ± 0.02 ^b^	4.03 ± 0.03 ^b^	3.77 ± 0.04 ^c^	3.81 ± 0.03 ^c^	3.80 ± 0.04 ^c^	0.133
C14:0	13.31 ± 0.09 ^a^	13.06 ± 0.07^b^	12.77 ± 0.1 ^cd^	12.91 ± 0.13 ^bc^	12.95 ± 0.09 ^bd^	12.63 ± 0.12 ^c^	0.0002
C16:0	32.05 ± 0.18 ^b^	31.45 ± 0.14 ^c^	30.9 ± 0.21 ^d^	32.97 ± 0.27 ^a^	32.52 ± 0.19 ^ab^	32.06 ± 0.24 ^b^	<0.0001
C18:0	13.03 ± 0.12 ^b^	12.62 ± 0.09 ^c^	12.35 ± 0.14 ^cd^	13.59 ± 0.18 ^a^	13.12 ± 0.13 ^bc^	12.82 ± 0.16 ^bc^	<0.0001
C18:1 *cis-*9	18.59 ± 0.18 ^d^	19.30 ± 0.15 ^bc^	19.65 ± 0.21 ^acb^	19.53 ± 0.28 ^b^	19.62 ± 0.2 ^b^	20.06 ± 0.24 ^a^	0.0009
Omega-6	1.66 ± 0.02 ^b^	1.73 ± 0.02 ^a^	1.77 ± 0.03 ^a^	1.58 ± 0.03 ^c^	1.61 ± 0.02 ^cb^	1.58 ± 0.03 ^cb^	0.0168
SCFA ^4^	8.33 ± 0.04 ^c^	8.39 ± 0.03 ^c^	8.51 ± 0.04 ^b^	8.60 ± 0.06 ^b^	8.72 ± 0.04 ^a^	8.81 ± 0.05 ^a^	0.0007
MCFA ^5^	21.13 ± 0.14 ^a^	20.78 ± 0.11 ^b^	20.39 ± 0.16 ^c^	20.06 ± 0.21 ^cd^	20.24 ± 0.15 ^cd^	19.88 ± 0.18 ^d^	0.0053
LCFA ^6^	65.33 ± 0.2 ^d^	65.12 ± 0.16 ^de^	64.67 ± 0.24 ^e^	67.68 ± 0.31 ^b^	66.88 ± 0.22 ^ac^	66.53 ± 0.27 ^c^	0.0048
Milk income (NZD/cow/day)	12.58 ± 0.26 ^b^	12.50 ± 0.21 ^b^	11.74 ± 0.30 ^c^	14.59 ± 0.39 ^a^	14.67 ± 0.28 ^a^	14.61 ± 0.34 ^a^	0.2208
SFC_10_^7^	69.83 ± 0.49 ^a^	67.49 ± 0.39 ^b^	65.03 ± 0.57 ^c^	70.45 ± 0.73 ^a^	69.21 ± 0.53 ^a^	66.89 ± 0.65 ^b^	<0.0001

^1^ Saturated fatty acids; ^2^ Unsaturated fatty acids; ^3^ Polyunsaturated fatty acids; ^4^ Short-chain fatty acids (sum of C4:0, C6:0 and C8:0); ^5^ Medium-chain fatty acids (sum of C10:0, C12:0); ^6^ Long-chain fatty acids (sum of C14:0, C16:0, C18:0 and C18:1 *cis*-9 and omega-6); ^7^ Solid fat content at 10 °C; ^8^ Milking frequency; Milk income was calculated using the equation NZD 9.78 × kg Fat yield + NZD 8.89 × kg Protein yield − NZD 0.033 × kg milk yield; ^a–f^ Means with different superscripts between genotypes in cows across milking frequency are significantly different (*p* < 0.05).

## Data Availability

Not applicable.

## References

[1] Davis S.R., Farr V.C., Stelwagen K. (1999). Regulation of yield loss and milk composition during once-daily milking: A review. Livest. Prod. Sci..

[2] Clark D.A., Phyn C.V.C., Tong M.J., Collis S.J., Dalley D.E. (2006). A systems comparison of once-versus twice daily milking of pastured dairy cows. J. Dairy Sci..

[3] Edwards J.P. (2019). Comparison of milk production and herd characteristics in New Zealand herds milked once or twice a day. Anim. Prod. Sci..

[4] Rémond B., Pomiès D., Dupont D., Chilliard Y. (2004). Once-a-day milking of multiparous Holstein cows throughout the entire lactation: Milk yield and composition, and nutritional status. Anim. Res..

[5] Lembeye F., López-Villalobos N., Burke J.L., Davis S.R. (2015). Estimation of breed and heterosis effects for milk traits and somatic cell scores in cows milked once and twice daily in New Zealand. Proc. N. Z. Soc. Anim. Prod..

[6] Sneddon N.W., Lopez-Villalobos N., Hickson R.E., Shalloo L., Garrick D.J. (2015). Estimation of crossbreeding effects on yields of dairy products and value of milk processed in different product portfolios. Proc. N. Z. Soc. Anim. Prod..

[7] Dalgleish D.G. (1993). Bovine milk protein properties and the manufacturing quality of milk. Livest. Prod. Sci..

[8] Walstra P., Wouters J.T., Geurts T.J. (2005). Dairy Science and Technology.

[9] MacGibbon A.K.H., McSweeney P.L.H., Fox P.F., O’Mahony J.A. (2020). Composition and Structure of Bovine Milk Lipids. Advanced Dairy Chemistry.

[10] Soyeurt H., Dardenne P., Gillon A., Croquet C., Vanderick S., Mayeres P., Bertozzi C., Gengler N. (2006). Variation in fatty acid contents of milk and milk fat within and across breeds. J. Dairy Sci..

[11] Palladino R.A., Buckley F., Prendiville R., Murphy J.J., Callan J., Kenny D.A. (2010). A comparison between Holstein-Friesian and Jersey dairy cows and their F1 hybrid on milk fatty acid composition under grazing conditions. J. Dairy Sci..

[12] Lopez-Villalobos N., Spelman R.J., Melis J., Davis S.R., Berry S.D., Lehnert K., Holroyd S.E., MacGibbon A.K., Snell R.G. (2014). Estimation of genetic and crossbreeding parameters of fatty acid concentrations in milk fat predicted by mid-infrared spectroscopy in New Zealand dairy cattle. J. Dairy Sci..

[13] Back P.J., Thomson N.A. (2005). Exploiting cow genotype to increase milk value through production of minor milk components. Proc. N. Z. Soc. Anim. Prod..

[14] Stoop W.M., Bovenhuis H., Heck J.M.L., van Arendonk J.A.M. (2009). Effect of lactation stage and energy status on milk fat composition of Holstein-Friesian cows. J. Dairy Sci..

[15] Palmquist D.L., Beaulieu A.D., Barbano D.M. (1993). Feed and animal factors influencing milk fat composition. J. Dairy Sci..

[16] Dewhurst R.J., Shingfield K.J., Lee M.R., Scollan N.D. (2006). Increasing the concentrations of beneficial polyunsaturated fatty acids in milk produced by dairy cows in high-forage systems. Anim. Feed Sci. Technol..

[17] Heck J.M., Van Valenberg H.J., Dijkstra J., Van Hooijdonk A.C. (2009). Seasonal variation in the Dutch bovine raw milk composition. J. Dairy Sci..

[18] Schwendel B.H., Morel P.C., Wester T.J., Tavendale M.H., Deadman C., Fong B., Shadbolt N.M., Thatcher A., Otter D.E. (2015). Fatty acid profile differs between organic and conventionally produced cow milk independent of season or milking time. J. Dairy Sci..

[19] MacGibbon A.K. (1996). Herd-to-herd variations in the properties of milkfat. Proc. N. Z. Soc. Anim. Prod..

[20] MacGibbon A.K., McLennan W.D. (1987). Hardness of New-Zealand patted butter-seasonal and regional variations. N. Z. J. Dairy Sci. Technol..

[21] Stoop W.M., Schennink A., Visker M.H.P.W., Mullaart E., Van Arendonk J.A.M., Bovenhuis H. (2009). Genome-wide scan for bovine milk-fat composition. I. Quantitative trait loci for short-and medium-chain fatty acids. J. Dairy Sci..

[22] Knutsen T.M., Olsen H.G., Tafintseva V., Svendsen M., Kohler A., Kent M.P., Lien S. (2018). Unravelling genetic variation underlying *de novo*-synthesis of bovine milk fatty acids. Sci. Rep..

[23] Spelman R.J., Ford C.A., McElhinney P., Gregory G.C., Snell R.G. (2002). Characterization of the DGAT1 gene in the New Zealand dairy population. J. Dairy Sci..

[24] Bovenhuis H., Visker M.H., Poulsen N.A., Sehested J., Van Valenberg H.J., Van Arendonk J.A., Larsen L.B., Buitenhuis A.J. (2016). Effects of the diacylglycerol o-acyltransferase 1 (DGAT1) K232A polymorphism on fatty acid, protein, and mineral composition of dairy cattle milk. J. Dairy Sci..

[25] Li Y., Zhou H., Cheng L., Edwards G.R., Hickford J.G. (2021). Effect of DGAT1 variant (K232A) on milk traits and milk fat composition in outdoor pasture-grazed dairy cattle. N. Z. J. Agric. Res..

[26] Schennink A., Stoop W.M., Visker M.W., Heck J.M., Bovenhuis H., Van Der Poel J.J., Van Valenberg H.J., Van Arendonk J.A. (2007). DGAT1 underlies large genetic variation in milk-fat composition of dairy cows. Anim. Genet..

[27] Tumino S., Criscione A., Moltisanti V., Marletta D., Bordonaro S., Avondo M., Valenti B. (2021). Feeding System Resizes the Effects of DGAT1 Polymorphism on Milk Traits and Fatty Acids Composition in Modicana Cows. Animals.

[28] Lopez-Villalobos N., Ariyarathne H.B.P.C., Gedye K., Correa-Luna M., Donaghy D.J. (2020). Association of a SNP in the DGAT1 gene with productive and reproductive performance and profitability in grazing cows milked once and twice a day. J. Dairy Sci..

[29] Sukhija P.S., Palmquist D.L. (1988). Rapid method for determination of total fatty acid content and composition of feedstuffs and feces. J. Agric. Food Chem..

[30] Wigginton J.E., Cutler D.J., Abecasis G.R. (2005). A note on exact tests of Hardy-Weinberg equilibrium. Am. J. Hum. Genet..

[31] Jiang J., Ma L., Prakapenka D., VanRaden P.M., Cole J.B., Da Y. (2019). A large-scale genome-wide association study in US Holstein cattle. Front. Genet..

[32] Grisart B., Farnir F., Karim L., Cambisano N., Kim J.J., Kvasz A., Mni M., Simon P., Frere J.M., Coppieters W. (2004). Genetic and functional confirmation of the causality of the DGAT1 K232A quantitative trait nucleotide in affecting milk yield and composition. Proc. Natl. Acad. Sci. USA.

[33] Lopez-Villalobos N., Spelman R.J., Melis J., Davis S.R., Berry S.D., Lehnert K., Sneddon N.W., Holroyd S.E., MacGibbon A.K., Snell R.G. (2020). Genetic correlations of milk fatty acid contents predicted from milk mid-infrared spectra in New Zealand dairy cattle. J. Dairy Sci..

[34] Soyeurt H., Gillon A., Vanderick S., Mayeres P., Bertozzi C., Gengler N. (2007). Estimation of heritability and genetic correlations for the major fatty acids in bovine milk. J. Dairy Sci..

[35] Marshall M.O., Knudsen J. (1979). Specificity of diacylglycerol acyltrans-ferase from bovine mammary gland, liver and adipose tissue towards acyl-CoA esters. Eur. J. Biochem..

[36] Ariyarathne H.B.P.C., Correa-Luna M., Blair H.T., Garrick D.J., Lopez-Villalobos N. (2021). Identification of genomic regions associated with concentrations of milk fat, protein, urea and efficiency of crude protein utilization in grazing dairy cows. Genes.

[37] Mackle T.R., Petch S.F., Bryant A.M., Auldist M.J., Henderson H.V., MacGibbon A.K.H. (1997). Variation in the characteristics of milkfat from pasture-fed dairy cows during late spring and the effects of grain supplementation. N. Z. J. Agric. Res..

[38] Kaylegian K.E. (1999). The production of specialty milk fat ingredients. J. Dairy Sci..

[39] Sanjayaranj I., Lopez-Villalobos N., Blair H.T., Janssen P.W.M., Holroyd S.E., MacGibbon A.K.H. (2022). Fatty Acid Composition of Dairy Milk: A Case Study Comparing Once- and Twice-a-Day Milking of Pasture-Fed Cows at Different Stages of Lactation. Dairy.

[40] Falconer D., Mackay M. (1996). Introduction to Quantitative Genetics.

[41] Lopez-Villalobos N. (2012). Analysing the genetic basis of milk production traits. CABI Rev..

[42] Delamaire E., Guinard-Flament J. (2006). Increasing milking intervals decreases the mammary blood flow and mammary uptake of nutrients in dairy cows. J. Dairy Sci..

[43] Bionaz M., Periasamy K., Rodriguez-Zas S.L., Hurley W.L., Loor J.J. (2012). A novel dynamic impact approach (DIA) for functional analysis of time-course omics studies: Validation using the bovine mammary transcriptome. PLoS ONE.

[44] Kęsek-Woźniak M., Wojtas E., Zielak-Steciwko A.E. (2020). Impact of SNPs in ACACA, SCD1, and DGAT1 genes on fatty acid profile in bovine milk with regard to lactation phases. Animals.

[45] Auldist M.J., Walsh B.J., Thomson N.A. (1998). Seasonal and lactational influences on bovine milk composition in New Zealand. J. Dairy Res..

